# 581. Impact of Urinary Antigen Tests on Clinical Outcomes in Patients Hospitalized with Community-acquired Pneumonia

**DOI:** 10.1093/ofid/ofad500.650

**Published:** 2023-11-27

**Authors:** Marita Kern, Simone Bastrup Israelsen, Thomas Benfield, Markus Fally, Pernille Ravn, Lilian Kolte

**Affiliations:** Copenhagen University Hospital - Hvidovre, Copenhagen, Sjelland, Denmark; Copenhagen University Hospital - Hvidovre, Copenhagen, Sjelland, Denmark; Copenhagen University Hospital, Hvidovre, Hovedstaden, Denmark; Copenhagen University Hospital – Bispebjerg and Frederiksberg, Copenhagen, Hovedstaden, Denmark; University of Copenhagen, Copenhagen, Hovedstaden, Denmark; Copenhagen University, Hilleroed, Sjelland, Denmark

## Abstract

**Background:**

Community-acquired pneumonia (CAP) is a common cause of hospital admission, with an annual incidence of 24.8 cases per 10,000 adults in the United States. To establish a microbiological diagnosis, most guidelines recommend using urinary antigen tests (UATs) in addition to respiratory samples in severe cases. However, it remains unclear how often these are used in clinical practice and with what impact. Therefore, we examined the impact of performing UATs on outcomes in patients hospitalized with CAP.

**Methods:**

This was a multicenter cohort study of immunocompetent adult patients admitted with CAP at three hospitals in Denmark from 2017 to 2020. The primary outcome was 30-day mortality, while broad-spectrum antibiotic treatment and coverage for atypical bacteria comprised secondary outcomes.

We used logistic regression to examine the impact of UATs on patient outcomes and applied propensity-score matching to adjust for potential confounders.

**Results:**

Of 2,449 patients included, 654 had UAT performed within 48 hours of admission. Of the tested group, 52/654 (8.0%) tested positive for S*treptococcus pneumoniae.*

30-day mortality was 11.8% in the tested group and 10.2% in the untested group (adjusted odds ratio [aOR] 1.17; 95% CI 0.83 - 1.65).

At discharge, 47.9% of the tested group received broad-spectrum antibiotics, compared with 42.4% in the untested group (aOR 1.25; 95% CI 1.00 - 1.55). Furthermore, 22.2% of the tested group had atypical coverage, compared with 21.9 % in the untested group (aOR 1.02; 95% CI 0.78 – 1.32).

Of patients with a positive pneumococcal UAT, 26.9% received broad-spectrum antibiotics at discharge, while 51.9% received broad-spectrum antibiotics in the UAT-negative group (aOR 0.34; 95% CI 0.15 – 0.77). Additionally, 21.2% of UAT-positive patients had atypical coverage at discharge, while 23.1% of UAT-negative patients had atypical coverage at discharge (aOR, 0.89; 95% CI, 0.35 – 2.26).

Forest plot for 30-day all-cause mortality

**Figure:**
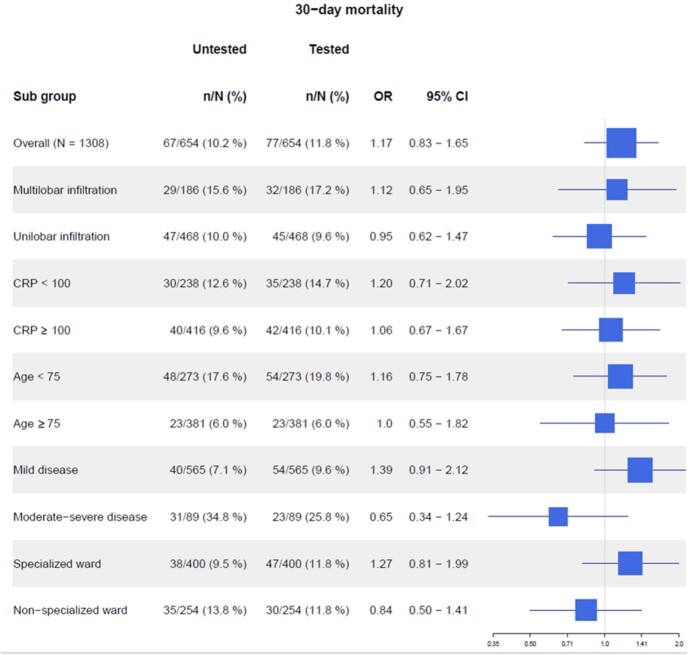

OR: Adjusted odds ratio; 95% CI: 95% confidence intervals; CRP: blood levels of C-reactive protein

Forest plot for treatment with broad-spectrum antibiotics at discharge

**Figure:**
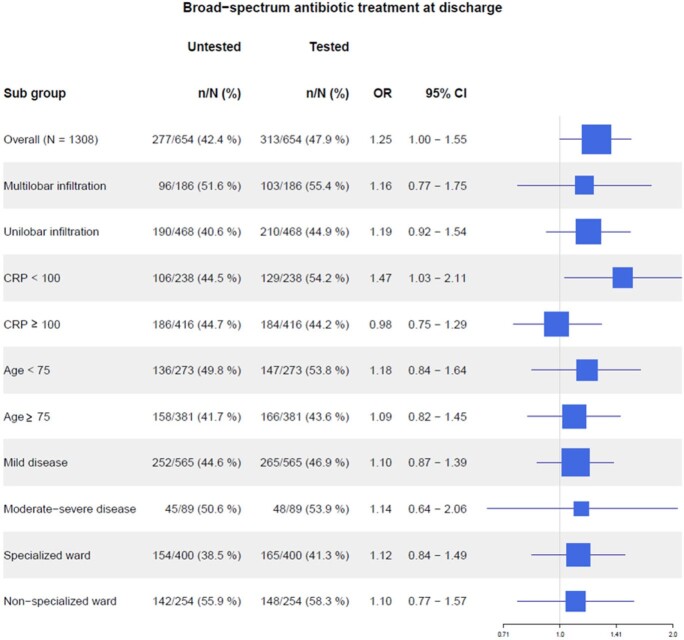

OR: Adjusted odds ratio; 95% CI: 95% confidence intervals; CRP: blood levels of C-reactive protein

Forest plot for coverage for atypical bacteria at discharge

**Figure:**
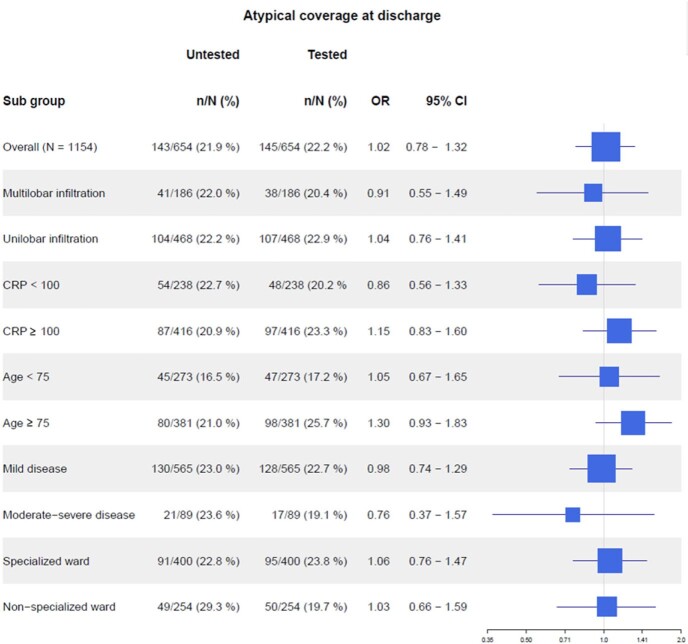

OR: Adjusted odds ratio; 95% CI: 95% confidence intervals; CRP: blood levels of C-reactive protein

**Conclusion:**

30-day mortality was similar between groups, whereas tested patients were slightly more likely to be treated with broad-spectrum antibiotics at discharge. Finally, patients with a UAT positive for *S. pneumoniae* less often received broad-spectrum antibiotics and coverage for atypical bacteria than UAT-negative patients at discharge.

**Disclosures:**

**All Authors**: No reported disclosures

